# Unlocking the healing power of Berberine: A promising aid for multiple sclerosis

**DOI:** 10.1016/j.ibneur.2026.06.007

**Published:** 2026-06-14

**Authors:** Amir Modarresi Chahardehi, Negar Karimi Khordeh, Narges Moazeni Limoudehi, Hediyeh Dasoomi, Razie Omrani, Mohammadhadi Nikjoo, Elham Mortazavi Mamaghani, Reza Nasiri, Mohammad Saeed Soleimani Meigoli, Fatemeh Shoja, Zahra Jafari-Ardakan, Reza Arefnezhad, Fatemeh Rezaei-Tazangi

**Affiliations:** a Student Research Committee, Fasa University of Medical Sciences, Fasa, Iran; b Student Research Committee, Kurdistan University of Medical Sciences, Sanandaj, Iran; c Student Research Committee, School of Paramedical and Rehabilitation Sciences, Mashhad University of Medical Sciences, Mashhad, Iran; d Student Research Committee, Ahvaz Jundishapur University of Medical Sciences, Ahvaz, Iran; e Institute of Cognitive and Brain Sciences, Shahid Beheshti University of Medical Sciences, Tehran, Iran; f Faculty of Medicine, Ahvaz Jundishapur University of Medical Sciences, Ahvaz, Iran; g Department of Medicine, Iran University of Medical Sciences, Tehran, Iran; h School of Medicine, Shiraz University of Medical Sciences, Shiraz, Iran; i Faculty of Medicine, Fasa University of Medical Sciences, Fasa, Iran; j Gerash University of Medical Sciences, Gerash, Iran; k Department of Basic Sciences, Faculty of Veterinary Medicine, University of Tehran, Tehran, Iran; l Student Research Committee, Shiraz University of Medical Sciences, Shiraz, Iran; m Department of Anatomy, School of Medicine, Fasa University of Medical Sciences, Fasa, Iran

**Keywords:** Berberine, Multiple sclerosis, Experimental autoimmune encephalomyelitis, Cuprizone model, Immunomodulation, Neuroprotection, Remyelination, Nanoformulations, JAK/STAT pathway, SPHK1/S1P pathway

## Abstract

Multiple sclerosis (MS) is a debilitating autoimmune disorder characterized by inflammatory demyelination and progressive neurodegeneration within the central nervous system (CNS). Despite advances in disease-modifying therapies (DMTs), current treatments primarily mitigate relapses and slow disease progression but fall short in comprehensively addressing cumulative disability or neurodegeneration. Berberine (BBR), a naturally occurring isoquinoline alkaloid, has emerged as a promising therapeutic candidate due to its potent immunomodulatory, anti-inflammatory, and neuroprotective properties. In this narrative review, we synthesize the molecular mechanisms underpinning BBR's effects on MS pathology and evaluate preclinical evidence from MS-relevant animal models. Studies in experimental autoimmune encephalomyelitis (EAE) —the primary MS model—and the cuprizone (CPZ) -induced demyelination model demonstrate that BBR (typically 5–300 mg/kg in preclinical protocols) reduces pro-inflammatory cytokines, modulates immune responses, and promotes remyelination—processes critical for counteracting MS-associated neurodegeneration. BBR modulates key signaling pathways, including JAK/STAT and SPHK1/S1P, which are pivotal in attenuating immune-mediated damage and preserving blood-brain barrier (BBB) integrity. Despite its therapeutic potential, challenges such as poor bioavailability and suboptimal pharmacokinetics have spurred investigations into advanced delivery systems. Nanoformulations, particularly BBR-loaded iron oxide nanoparticles (BBR-IONP), have shown superior efficacy in preclinical models by enhancing CNS delivery and improving remyelination outcomes. By highlighting BBR's multifaceted bioactivities, this review underscores its promise as a complementary or alternative approach to address unmet needs in MS management, while acknowledging the critical need for clinical trials to validate these preclinical findings.

## Graphical Abstract

**Figure:**
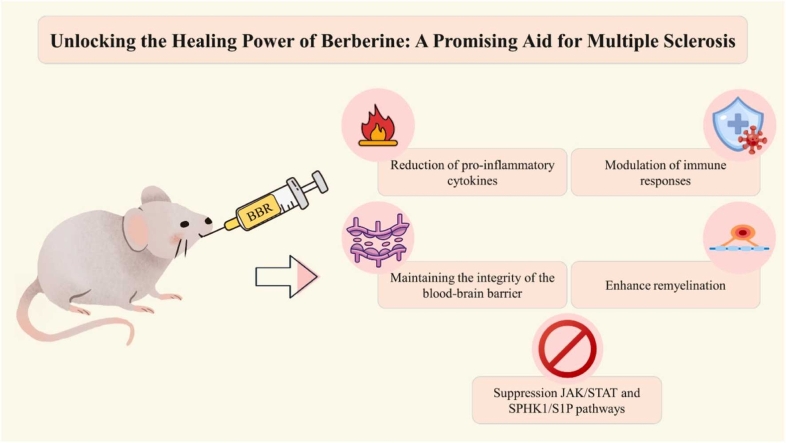
Berberine exerts anti-multiple sclerosis effects primarily by attenuating inflammatory traction, orchestrating immune reactions, promoting remyelination, maintaining blood-brain barrier integrity, and regulating involved signaling pathways.

## 1 Introduction

Multiple sclerosis (MS) is a chronic autoimmune disorder of the central nervous system (CNS), characterized by inflammatory demyelination and progressive neurodegeneration, resulting in lesions affecting both gray and white matter (Travers et al., 2022). This complex condition manifests through a spectrum of symptoms, including fatigue, motor dysfunction, sensory impairments, spasticity, cognitive deficits, and urinary dysfunction, significantly impacting patients’ quality of life (Arneth, 2024, Piacentine et al., 2024). With a global prevalence exceeding 2.8 million individuals, MS disproportionately affects young adults, particularly women, and exhibits a higher incidence in regions farther from the equator (Ostolaza et al., 2021, Vaughn et al., 2019). Diagnosis relies on clinical presentation, often supplemented by paraclinical tools such as magnetic resonance imaging (MRI) and cerebrospinal fluid analysis, to confirm inflammatory and demyelinating activity (Oh, 2022). MS presents in various forms, including relapsing-remitting MS (RRMS) and progressive subtypes, each posing unique therapeutic challenges (Sevastou et al., 2016).

Current management strategies for MS focus on three pillars: treating acute relapses, slowing disease progression with disease-modifying therapies (DMTs), and alleviating symptoms to improve functionality (Haki et al., 2024). While DMTs, such as interferon-beta, fingolimod, and monoclonal antibodies, effectively reduce relapse rates and delay disease progression, they fall short in halting long-term disability or addressing neurodegeneration (Vaughn et al., 2019). Moreover, challenges such as treatment adherence, management of comorbidities, and equitable access to care—particularly in resource-limited settings—continue to hinder optimal outcomes (Yazdanpanah et al., 2024). These limitations underscore the urgent need for novel therapeutic agents that can complement existing treatments and target both inflammatory and neurodegenerative components of MS.

**Fig. 1:**
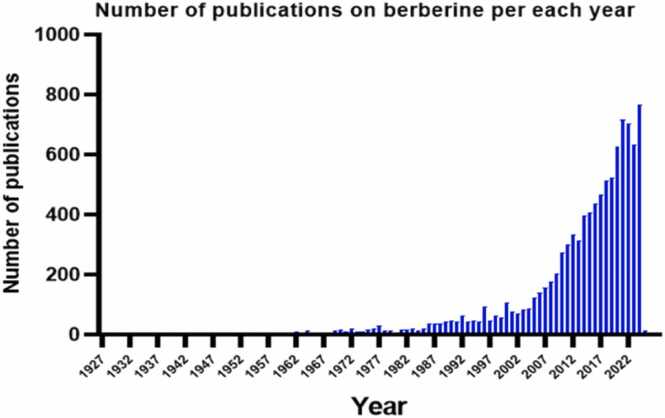
Number of publications mentioning Berberine from 1927 to December 2024 according to PubMed.

Berberine (BBR), a naturally occurring isoquinoline alkaloid derived from plants such as Berberis vulgaris and *Coptis chinensis*, has emerged as a promising candidate due to its multifaceted pharmacological properties, including anti-inflammatory, antioxidant, and immunomodulatory effects (Li et al., 2023). While extensively studied across various conditions like metabolic and inflammatory disorders (Tavaf et al., 2023, Ashrafizadeh et al., 2020), its relevance to MS stems from its direct effects on the CNS. In preclinical models, particularly experimental autoimmune encephalomyelitis (EAE), BBR reduces disease severity by suppressing pro-inflammatory cytokines (e.g., IL−1β, IL−6, TNF-α), enhancing regulatory T-cell (Treg) populations, and promoting anti-inflammatory mediators like interleukin−10 (IL−10) (Ignacio et al., 2020, Thompson et al., 2018). These effects are mediated through the modulation of key signaling pathways such as NF-κB, JAK/STAT, and SPHK1/S1P (Ashrafizadeh et al., 2020), which are central to MS pathogenesis. Despite its therapeutic promise, BBR's clinical application is limited by poor bioavailability, prompting research into advanced delivery systems like nanoformulations to enhance its pharmacokinetic profile (Och et al., 2020). The growing interest in BBR is evidenced by over 766 publications in 2024 alone (PubMed, Fig. 1).

The intersection of BBR and MS therapy is particularly compelling, as its immunomodulatory and neuroprotective properties align with the unmet needs in MS management. By attenuating inflammation, promoting remyelination, and protecting neuronal integrity, BBR holds potential as an adjunctive or standalone therapy to enhance the efficacy of conventional DMTs. This narrative review specifically focuses on the potential role of BBR in multiple sclerosis. Rather than providing a general overview of Berberine pharmacology, we aim to synthesize MS-relevant preclinical evidence, particularly from EAE and cuprizone (CPZ) models, and to organize current knowledge according to key pathological processes in MS, including immune dysregulation, blood–brain barrier disruption, demyelination, and neurodegeneration. By integrating mechanistic and experimental data, this review seeks to clarify whether Berberine represents a biologically plausible adjunctive strategy in MS management.

## 2 Understanding multiple sclerosis

MS is a chronic, immune-mediated disorder of the CNS characterized by inflammation, demyelination, and neurodegeneration (Ganesan et al., 2023). The pathophysiology involves an autoimmune attack on the myelin sheath, driven by autoreactive T and B cells that infiltrate the CNS, leading to axonal injury and neuronal loss (Nicholas and Rashid, 2013, Dighriri et al., 2023, Cree et al., 2022). Also, it involves a complex interplay of genetic susceptibility, most notably within the HLA-DRB1 gene, and environmental triggers such as Epstein-Barr virus infection, vitamin D deficiency, and smoking (Haki et al., 2024, Dighriri et al., 2023, Cree et al., 2022). These factors contribute to a breakdown in immune tolerance, leading to an autoimmune attack on the myelin sheath. This process is primarily driven by autoreactive T cells (particularly Th1 and Th17 subtypes) and B cells, which infiltrate the CNS, promote inflammation, and cause axonal injury and neuronal loss, as summarized in Table 1 (Nicholas and Rashid, 2013, Dighriri et al., 2023, Cree et al., 2022, Tawfeeq et al., 2022).

**Table 1:** Principal pathophysiological characteristics of MS.

**Feature**	**Characteristic**
Immune dysregulation	Autoimmune attack on myelin facilitated by T and B cells
Genetic predisposition	Association with HLA-DRB1 and other genetic loci
Environmental triggers	EB infection, vitamin D deficiency, and smoking
Neurodegeneration	The debilitating effects of axonal loss and chronic active plaques

The clinical manifestations of MS are diverse and depend on the location and extent of CNS lesions. Common symptoms include motor weakness, spasticity, sensory disturbances, visual impairment (e.g., optic neuritis), cognitive dysfunction, and debilitating fatigue, all of which significantly impact patients' quality of life (Tafti et al., n.d, Correia et al., 2024, Pogoda-Wesołowska et al., 2023, Yi et al., 2024).

The disease course is heterogeneous and is typically classified into three main phenotypes. Relapsing-remitting MS (RRMS), the most common form at onset (∼85% of patients), is characterized by episodic neurological worsening (relapses) followed by periods of partial or full recovery (Cree et al., 2022, Inglese, 2006, Ford, 2020). Over time, many RRMS patients transition to secondary-progressive MS (SPMS), a phase marked by a steady accumulation of disability independent of relapses, driven by chronic neurodegeneration and axonal loss (Koch et al., 2022, Klineova and Lublin, 2018, Barcutean et al., 2024, Tullman, 2013, Cree et al., 2021). In contrast, primary-progressive MS (PPMS), affecting 10–15% of patients, is defined by continuous disease progression from onset without distinct relapses (Cree et al., 2022, Inglese, 2006, Rojas et al., 2018, Raghavan et al., 2015).

## 3 The therapeutic potential of Berberine

**Fig. 2:**
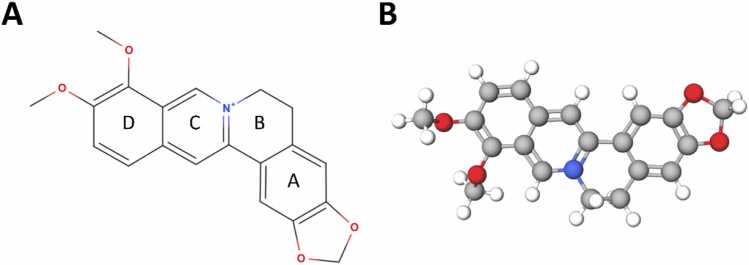
A) Molecular structure of BBR (2D), and B) 3D model (drawn with Molview.org).

Berberine (C_20_H_18_NO_4_^+^, IUPAC name: 16,17-dimethoxy−5,7-dioxa−13-azoniapentacyclo [11.8.0.0^2,10^.0^4,8^.0^15,20^] henicosa−1(13),2,4(8),9,14,16,18,20-octaene, PubChem CID: 2353) (Cheng et al., 2022), an alkaloid containing benzylisoquinolines found in nature (Ai et al., 2021). The molecular structure of BBR consists of a planar ring system with a quaternary ammonium group, which is essential for its biological activity (Fig. 2) (Ai et al., 2021). With a long history of use in traditional Chinese, Iranian, and Ayurvedic medicine for metabolic and gastrointestinal disorders, BBR has garnered significant scientific interest for its broad spectrum of pharmacological activities (Yazdanpanah et al., 2024, Qin et al., 2024).

The therapeutic potential of BBR stems from its potent anti-inflammatory, antioxidant, and immunomodulatory properties (Qin et al., 2024). Its anti-inflammatory effects are mediated through the suppression of pro-inflammatory cytokines such as tumor necrosis factor-alpha (TNF-α), interleukin−1β (IL−1β), and interleukin−6 (IL−6), while simultaneously promoting anti-inflammatory mediators like interleukin−10 (IL−10) (Cheng et al., 2022, Choi et al., 2006, Shou and Shaw, 2022). BBR also inhibits key inflammatory pathways, including NF-κB and mitogen-activated protein kinase (MAPK), thereby reducing the expression of cyclooxygenase−2 (COX−2) and matrix metalloproteinases (MMPs) (Ma et al., 2010, Kuo et al., 2012).

From an immunomodulatory perspective, BBR exerts regulatory effects on T-cell differentiation and function. It suppresses the development of pro-inflammatory Th1 and Th17 cells, which play pivotal roles in autoimmune pathogenesis, while enhancing the population and suppressive function of Tregs (Cui et al., 2009, Li et al., 2022). This ability to rebalance the Th17/Treg axis has been demonstrated in various experimental autoimmune models, including myasthenia gravis, rheumatoid arthritis, and uveitis (Song et al., 2022, Vita et al., 2021, Du et al., 2020, Liu et al., 2016).

Importantly for CNS disorders like MS, BBR can cross the blood-brain barrier (BBB) and exert direct neuroprotective effects within the central nervous system (Och et al., 2020, Luo et al., 2017). These effects include the inhibition of microglial activation, reduction of oxidative stress through activation of the nuclear factor erythroid 2-related factor 2 (Nrf2)/ heme oxygenase−1 (HO−1) antioxidant pathway, and protection against neuronal apoptosis (Cheng et al., 2022, Sunhe et al., 2024, Han et al., 2012). Furthermore, BBR's ability to preserve BBB integrity by reducing matrix metalloproteinase−9 (MMP−9) activity is particularly relevant to MS pathophysiology, where BBB disruption facilitates inflammatory cell infiltration into the CNS (Fuzimoto and Brigo, 2020, Jiang et al., 2013). These properties form the basis for its potential therapeutic application in inflammatory and neurodegenerative disorders, including multiple sclerosis.

### 3.1 Bioactive properties of Berberine

While this review focuses on MS, understanding BBR's general bioactive properties—derived from studies in both autoimmune and non-autoimmune conditions—provides important context for its potential mechanisms in MS. The following discussion draws on evidence from various disease models to illustrate BBR's anti-inflammatory, immunomodulatory, and neuroprotective effects, which are directly relevant to MS pathophysiology. However, it should be noted that clinical evidence discussed in this section derives from non-MS conditions and should not be misinterpreted as MS-specific data. BBR is acknowledged for its anti-inflammatory, antioxidant, and neuroprotective properties. It has been demonstrated to significantly diminish oxidative stress and inflammation, which are pivotal contributors in the pathophysiology of several neurological illnesses (Qin et al., 2024). BBR’s influence on immune modulation has been extensively studied in both autoimmune and non-autoimmune diseases. Numerous investigations have highlighted its potential to mitigate inflammation associated with autoimmune conditions (Wang et al., 2020). Th17 and Th1 are two subsets of inflammatory T cells that play pivotal roles in the development of various autoimmune diseases by secreting IFN-γ and IL−17, respectively (Cui et al., 2009). Conversely, CD4 + Foxp3 + Treg cells are critical for maintaining immune tolerance and regulating excessive inflammatory and immune responses triggered by Th cells (Li et al., 2022). BBR has demonstrated the ability to regulate inflammatory responses by suppressing pro-inflammatory cytokines, including tumor necrosis factor-alpha (TNF-α) and interleukin−6 (IL−6). This regulation is essential in disorders marked by persistent inflammation, such as autoimmune diseases (Cheng et al., 2022). BBR specifically elevates interleukin−10 (IL−10) levels, a cytokine possessing anti-inflammatory characteristics, while concurrently downregulating NF-κB, a crucial component in inflammatory reactions (Shou and Shaw, 2022).

In 1986, the earliest research on BBRs' cytotoxic effects was published (Wang et al., 1986). Dosage and duration determine BBR's cytotoxic effects on cancer cell lines. Numerous mechanisms of BBR's anticancer action have been uncovered by research. In a time- and dose-dependent manner, 33 genes involved in cell cycle, differentiation, and epithelial-mesenchymal transition were downregulated after BBR treatment in the human colon adenocarcinoma HCA−7 cell line, according to an assessment of 44 genes' expression levels (Och et al., 2020). BBR's anti-brain-tumor effects are mostly attributable to its ability to prevent tumor growth through mechanisms such as suppression of angiogenesis and induction of cell death. Furthermore, BBR enhances the effectiveness of chemotherapy, demonstrating a synergistic effect (Shou and Shaw, 2022).

A study using experimental autoimmune myasthenia gravis (EAMG) rat models demonstrated that oral administration of BBR significantly reduced disease severity and restored T cell (Th1, Th17, and Th1/17 subsets) homeostasis. This effect was attributed to the inhibition of the Janus Kinase (JAK)/STAT signaling pathway through molecular interactions between BBR and JAK2, thereby rebalancing Th cell subsets (Song et al., 2022). In another study, BBR was administered to mice before the onset of collagen-induced arthritis (CIA), demonstrating prophylactic efficacy with an arthritis incidence of 50%, compared to 90% in control groups. BBR delayed arthritis onset by decreasing T cell populations, particularly CD4 + CXCR5 + Tfh and CD4 + Th cells, while increasing Foxp3 + Treg cells. Additionally, it reduced the expression of co-stimulatory molecules in T cells and lowered anti-CII antibody concentrations (Vita et al., 2021). Du *et al*. explored the mechanisms by which BBR ameliorates experimental autoimmune uveitis (EAU). Clinical and histological scores indicated that BBR prevented the breakdown of the blood-retinal barrier (BRB). In the spleens of BBR-treated mice, reductions in Th1 and Th17 cells were observed, alongside an increase in Treg cell frequency. RNA sequencing of spleen samples revealed gene expression changes linked to immune pathways and chromatin remodeling. Furthermore, BBR altered the gut microbiota composition in EAU mice, increasing bacteria with immunomodulatory properties (Du et al., 2020).

BBR’s immunomodulatory properties were also observed in experimental autoimmune myocarditis (EAM). EAM caused an increase in Th1 and Th17 cells and their associated cytokines (IFN-γ and IL−17). However, BBR effectively restored immune balance and reduced the overexpression of phosphorylated proteins involved in the JAK-STAT pathway (p-STAT1, p-STAT3, and p-STAT4) (Liu et al., 2016). In non-autoimmune diseases, BBR’s anti-inflammatory effects may alleviate conditions like schizophrenia, where increased immune and inflammatory responses contribute to negative symptoms (Li et al., 2022). Dendritic cells (DCs), essential antigen-presenting cells that bridge adaptive and innate immune responses, are also targets of BBR. Studies have shown that BBR decreases the survival and inflammatory functions of DCs by inducing apoptosis, inhibiting co-stimulatory molecule expression, and reducing inflammatory cytokine secretion (Ehteshamfar et al., 2020).

BBR’s neuroprotective effects are partly attributed to its immunomodulatory properties (Kumar et al., 2015). For example, diabetes mellitus’s propensity to increase the risk of CNS disorders such as seizures, stroke, cognitive impairment, and dementia prompted an investigation into BBR’s impact on glial cell reactivity in the hippocampus of diabetic rats. The findings revealed that BBR treatment prevented an increase in astrocytes expressing glial fibrillary acidic protein (GFAP) and reduced the number of GFAP-immunoreactive astrocytes in the hippocampus (Moghaddam et al., 2014). Moreover, BBR's capacity to traverse the BBB enables it to directly influence the central nervous system, positioning it as a potential therapeutic agent for neurodegenerative disorders (Och et al., 2020).

## 4 BBR in multiple sclerosis: Mechanistic and preclinical evidence

Preclinical studies have evaluated BBR in two complementary animal models of MS: EAE, which recapitulates immune-mediated aspects, and the CPZ model, which isolates demyelination and remyelination processes. Building on the general pharmacological properties outlined in 3, this section integrates all MS-relevant mechanisms into a unified framework. Key findings are systematically summarized in Table 2, with mechanisms of action detailed below.

**Table 2:** Neuroprotective and anti-inflammatory effects of BBR in experimental models of MS.

**Model**	**Protocol type**	**Dose/Route**	**Timing**	**Key mechanisms**	**CNS outcomes**	**Peripheral outcomes**	**Ref.**
EAE (C57BL/6 mice)	Therapeutic	10, 30 mg/kg (oral)	Days 3–28 post-immunization	Th1/Th17 suppression, Treg/Th2 enhancement	↓ Demyelination (spinal cord); ↓ inflammatory infiltration	↓ IFN-γ, TNF-α, IL−17 (splenocytes); ↑ IL−4, IL−10, IL−35	(Tavaf et al., 2023)
EAE (C57BL/6 mice)	Therapeutic	50, 100, 300 mg/kg (oral)	Days 3–28 post-immunization	SphK1/S1P inhibition	↓ Demyelination; ↓ neurophysiological deficits; ↓ SphK1/S1P in spinal cord	↓ SphK1/S1P in splenocytes	(Luo et al., 2017)
EAE (C57BL/6 mice)	Prophylactic & Therapeutic	5, 10, 20, 50, 100, 200 mg/kg (i.p.)	Days −1–28 (prophylactic) or days 10–28 (therapeutic)	JAK/STAT inhibition, Th1/Th17 suppression	↓ Clinical scores; ↓ inflammatory infiltration	↓ IL−6 production by APCs; ↓ Th1/Th17 differentiation	(Qin et al., 2010)
EAE (C57BL/6 mice)	Therapeutic	Not specified	Days 7–28 post-immunization	MMP−9 inhibition, laminin protection	↓ TUNEL + neurons; ↓ gelatinase activity; ↓ laminin degradation	Not assessed	(Jiang et al., 2013)
CPZ-induced neurotoxicity (rats)	Therapeutic	50 mg/kg/day free BBR and BBR-IONP (oral)	Weeks 0–5 (concurrent with CPZ)	Anti-inflammatory, antioxidant, remyelination	↑ MBP; ↓ GFAP, S100β (cortex, hippocampus); cognitive improvement	↓ TNF-α, MMP−9 (serum); ↑ GSH (serum)	(Ibrahim Fouad et al., 2024)

### 4.1 Immunomodulation and T-cell regulation

The immunomodulatory effects of BBR have been demonstrated primarily in EAE models using both prophylactic and therapeutic protocols. Qin et al. (2010) showed that BBR administration (5–200 mg/kg i.p.) initiated either before immunization prophylactic) or after disease onset (therapeutic) significantly reduced clinical severity. This was associated with suppression of Th1 and Th17 differentiation in peripheral lymphoid organs, mediated through inhibition of JAK/STAT signaling. Similarly, Tavaf et al. (2023) reported that BBR treatment (10–30 mg/kg oral) reduced pro-inflammatory cytokines (IFN-γ, TNF-α, IL−17) while enhancing anti-inflammatory mediators (IL−4, IL−10, IL−35) and Treg populations. These findings establish immunomodulation as a central mechanism by which BBR attenuates CNS autoimmunity.

### 4.2 Preservation of blood-brain barrier integrity

BBR preserves BBB integrity through inhibition of MMP−9. Jiang et al. (2013) demonstrated that therapeutic BBR treatment in EAE mice reduced MMP−9 activity in the CNS, decreased gelatinase activity, and prevented laminin degradation within the brain parenchyma. These effects correlated with reduced TUNEL-positive neuronal cells, indicating that BBB preservation contributes to neuroprotection by limiting inflammatory infiltration.

### 4.3 Inhibition of the SphK1/S1P signaling pathway

The SphK1/S1P pathway, which is upregulated in MS, represents another key target of BBR. Luo et al. (2017) demonstrated that BBR treatment (50–300 mg/kg oral) in EAE mice dose-dependently reduced SphK1 activity and S1P levels in both spinal cord and splenocytes, associated with reduced demyelination, improved neurophysiological function, and decreased astrocyte activation. This pathway modulation affects both the CNS and peripheral compartments.

### 4.4 Microglial modulation and neuroinflammation

**Fig. 3:**
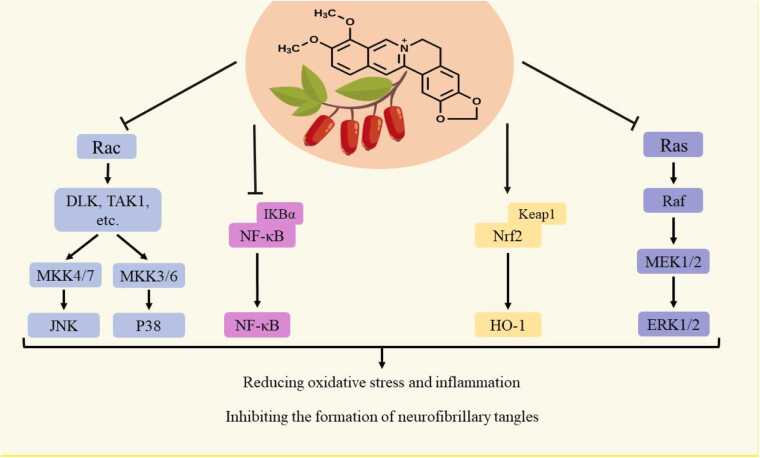
Berberine's mechanisms for enhancing neural repair and modulating neuroinflammation. The figure illustrates key signaling pathways involved in BBR's neuroprotective effects, including inhibition of NF-κB (via IκBα stabilization), activation of Nrf2/HO−1 antioxidant responses, and modulation of MAPK pathways (JNK, ERK). These mechanisms contribute to reduced microglial activation, decreased inflammatory mediator production, and enhanced neuronal survival in the context of MS-associated neuroinflammation. Abbreviations: IKBα (Inhibitor of κB alpha), NF-κB (Nuclear Factor Kappa B), Keap1 (Kelch-like ECH-associated protein 1), Nrf2 (Transcription factor Nrf2), HO−1 (Heme oxygenase 1), DLK (Dual leucine zipper kinase), JNK (C-Jun N-Terminal Kinases), ERK (extracellular signal-regulated kinase).

Building on the general anti-inflammatory properties introduced in 3, BBR exerts neuroprotective effects relevant to neural repair through modulation of microglial activation and enhancement of antioxidant defenses. As illustrated in Fig. 3, BBR inhibits microglial activation and reduces production of inflammatory mediators (TNF-α, IL−1β) through NF-κB and MAPK pathways (Cheng et al., 2022, Sunhe et al., 2024). Fig. 3 summarizes the key signaling pathways involved in BBR's neuroprotective effects, including inhibition of NF-κB (via IκBα stabilization), activation of Nrf2/HO−1 antioxidant responses, and modulation of MAPK pathways (JNK, ERK). Concurrently, BBR activates the Nrf2/HO−1 pathway in neural cells, reducing oxidative stress and supporting mitochondrial function (Cheng et al., 2022, Han et al., 2012). These mechanisms create a favorable environment for neuronal survival and subsequent repair processes.

In the CPZ model, Ibrahim Fouad et al. (2024) showed that BBR treatment reduced astrogliosis (GFAP, S100β) and attenuated neuroinflammation, consistent with the microglial modulatory effects described above and illustrated in Fig. 3.

### 4.5 Remyelination and oligodendrocyte precursor cell effects

Beyond its neuroprotective properties, BBR significantly contributes to remyelination through direct effects on oligodendrocyte precursor cells (OPCs). Studies demonstrate that BBR improves the viability and development of OPCs, enhancing their proliferation and differentiation into mature myelinating oligodendrocytes (Han et al., 2012). In peripheral nerve injury models, BBR treatment increased the thickness of remyelinated axons and promoted neurite outgrowth (Cheng et al., 2022).

Direct evidence for BBR's pro-remyelinating effects in MS models has been provided by several studies. In the CPZ model, Ibrahim Fouad et al. (2024) demonstrated that BBR treatment significantly increased myelin basic protein (MBP) expression and promoted OPC proliferation and differentiation, with nanoformulated BBR BBR-IONP) showing superior efficacy. In the EAE model, Luo et al. (2017) showed that BBR preserved axonal integrity as demonstrated by decreased SMI−32 positivity and increased MBP staining in spinal cord sections. These findings indicate that BBR not only prevents demyelination but also actively supports regenerative processes.

### 4.6 Integration of mechanisms: a multi-targeted approach

**Fig. 4:**
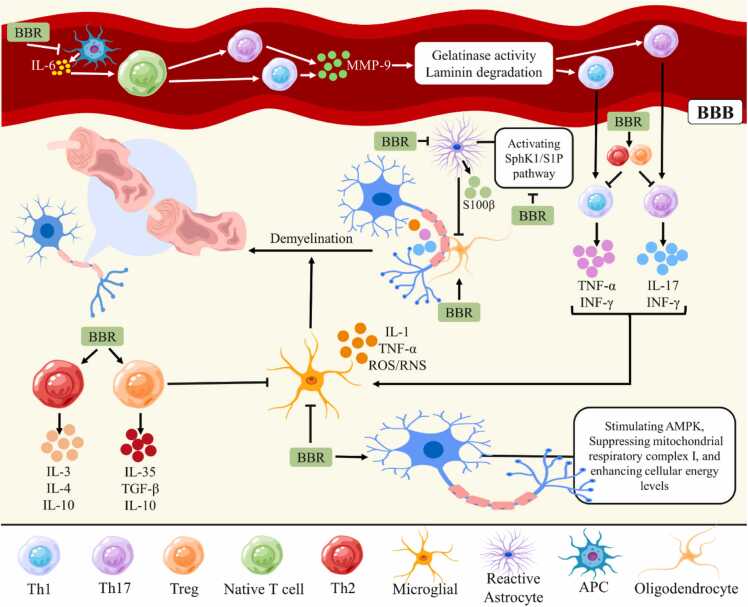
Berberine's multi-targeted mechanisms of action in multiple sclerosis. This figure integrates all MS-relevant pathways discussed in 4, illustrating how BBR simultaneously targets immune dysregulation (Th1/Th17 suppression, Treg enhancement), blood-brain barrier integrity (MMP−9 inhibition), neuroinflammation (microglial modulation), demyelination (SphK1/S1P pathway inhibition), and oxidative stress (Nrf2/HO−1 activation). Abbreviations: Th (T helper), Treg (Regulatory T cells), BBR (Berberine), APC (antigen-presenting cell), IL (Interleukin), TGF-β (Transforming growth factor beta), AMPK (AMP-activated protein kinase), ROS (Reactive oxygen species), RNS (Reactive nitrogen species), INF-γ (Interferon gamma), TNF-α (Tumor necrosis factor alpha), BBB (blood–brain barrier), MMP−9 (Matrix metallopeptidase 9), SphK1/S1P (Sphingosine Kinase 1/Sphingosine−1-phosphate), S100β (S100 calcium-binding protein β).

The mechanisms outlined above do not operate in isolation but form an integrated therapeutic network, as summarized in Fig. 4. Immunomodulation in peripheral compartments (4.1) reduces the inflammatory drive that initiates CNS damage through suppression of Th1 and Th17 cells and enhancement of Treg populations (Tavaf et al., 2023, Qin et al., 2010). Preservation of BBB integrity (4.2) limits entry of pathogenic immune cells by inhibiting MMP−9 activity and preventing laminin degradation (Jiang et al., 2013). Within the CNS, SphK1/S1P inhibition (4.3) reduces demyelination and astrocyte activation (Luo et al., 2017), while microglial modulation (4.4) attenuates local neuroinflammation through NF-κB and MAPK pathways (detailed in Fig. 3) (Cheng et al., 2022, Sunhe et al., 2024). Concurrently, direct effects on OPCs (4.5) promote remyelination and axonal preservation through enhanced MBP expression and reduced axonal damage (Luo et al., 2017, Ibrahim Fouad et al., 2024). Fig. 4 provides a comprehensive visualization of these interconnected mechanisms, illustrating how BBR simultaneously targets multiple pathophysiological features of MS—including immune dysregulation, BBB disruption, neuroinflammation, demyelination, and impaired remyelination. This multi-targeted profile distinguishes BBR from conventional disease-modifying therapies that typically target pathways and positions it as a promising candidate for comprehensive MS management.

### 4.7 Translational challenges and the absence of MS-specific clinical evidence

It is critical to note that, despite extensive preclinical research, no clinical trials have investigated BBR in patients with multiple sclerosis. While BBR has been evaluated in human studies for other conditions—including type 2 diabetes, hyperlipidemia, metabolic syndrome, and schizophrenia (Zhang et al., 2020; Li et al., 2015; Pu et al., 2023)—these findings cannot be directly extrapolated to MS. The discussion of non-MS clinical studies in this section is intended solely to illustrate the general safety profile and bioavailability challenges of BBR in humans, not to imply evidence of efficacy in MS.

#### 4.7.1 Lack of human studies in MS

To date, no clinical trials have specifically evaluated BBR in patients with multiple sclerosis. While BBR has been investigated in human studies for other conditions—including type 2 diabetes, hyperlipidemia, and metabolic syndrome (Zhang et al., 2020; Li et al., 2015)—its effects on MS-related outcomes such as relapse rate, disability progression, or MRI measures of disease activity remain unexplored. This represents a significant gap in the evidence base.

#### 4.7.2 Bioavailability as a primary translational barrier

The most formidable obstacle to clinical translation is BBR's poor oral bioavailability (<1%), resulting from low intestinal absorption, extensive first-pass metabolism, and rapid systemic elimination (Och et al., 2020). After oral administration, BBR is poorly absorbed in the upper gastrointestinal tract and is actively effluxed by P-glycoprotein back into the intestinal lumen. The fraction that is absorbed undergoes rapid metabolism in the liver, primarily by cytochrome P450 enzymes (CYP2D6, CYP3A4), yielding metabolites with reduced biological activity (Ai et al., 2021, Qin et al., 2024). Consequently, achieving therapeutic concentrations within the CNS—where BBR must act to exert its neuroprotective and remyelination-promoting effects—remains a substantial challenge.

Recent clinical pharmacokinetic data have further elucidated the complexity of BBR metabolism. Blocher et al. (2025) demonstrated that CYP2D6 polymorphisms significantly affect BBR metabolism in a sex-dependent manner, with females showing 2.8-fold higher area under the curve (AUC) compared to males. This finding has vital implications for personalized dosing strategies and highlights the need for careful consideration of patient characteristics in future clinical trials.

#### 4.7.3 Emerging solutions, nanoformulations, and delivery systems

Beyond BBR-IONP, which has been directly evaluated in the CPZ model (Ibrahim Fouad et al., 2024), several other nanoformulation strategies have been developed to overcome BBR's pharmacokinetic limitations, though they await testing in MS-specific models.

Liposomal formulations have been shown to enhance BBR's oral bioavailability by protecting it from degradation in the gastrointestinal tract and improving intestinal absorption. Studies in non-MS models demonstrate that liposomal encapsulation increases BBR's circulation time and facilitates targeted delivery to inflamed tissues (Chaudhari et al., 2025). Polymeric nanoparticles, particularly those composed of PLGA (poly(lactic-co-glycolic acid)) and chitosan, offer sustained release profiles and improved brain penetration. PLGA nanoparticles loaded with BBR have demonstrated enhanced neuroprotective effects in models of cerebral ischemia and Parkinson's disease, with improved BBB penetration and reduced oxidative stress (Bogdanova et al., 2025).

A landmark study by Bian et al. (2025) introduced a BBR-inspired ionizable lipid platform (BE-ST) for brain-targeted nucleic acid delivery. By leveraging BBR's affinity for dopamine D3 receptors (highly expressed in brain regions affected by neurodegenerative diseases), these nanoparticles achieved efficient BBB penetration and brain-specific accumulation, demonstrating therapeutic efficacy in mouse models of Alzheimer's disease and glioma. On the other hand, a recent comprehensive review by Pal et al. (2025) summarized the translational potential of BBR-based nanopreparations for neurodegenerative diseases, emphasizing that future nanocarrier development and mechanistic investigation will be essential for next-generation therapeutics. While BBR-IONP remains the only nanoformulation evaluated in MS-specific models to date (Ibrahim Fouad et al., 2024), these advances in related neurodegenerative disease models provide a strong foundation for future research. While these diverse nanoformulation strategies have shown promise in related neurodegenerative disease models, their evaluation in MS-specific models (EAE and CPZ) remains a critical gap. Systematic comparison of these approaches—including BBR-IONP, liposomal, polymeric, phytosomal, and lipid-based formulations—in head-to-head preclinical studies represents an essential next step toward clinical translation.

#### 4.7.4 Insights from clinical trials in other neurological disorders

Although MS-specific data are lacking, clinical evidence from other CNS disorders provides valuable insights into BBR's potential effects on neurological function and its safety profile in human populations.

In Alzheimer's disease, extensive preclinical studies have demonstrated that BBR (5–260 mg/kg in animal models) reduces amyloid-beta deposits, decreases tau hyperphosphorylation, and improves cognitive function in AD transgenic mice through modulation of multiple pathways, including PI3K/Akt, Nrf2/HO−1, and AMPK signaling pathways (Dan et al., 2023, Begh et al., 2025). A systematic review and meta-analysis by Dan et al. (2023) confirmed that BBR significantly shortened escape latency, increased platform crossings, and decreased Aβ1–42 deposition and tau phosphorylation across 22 preclinical studies, with the protective effects closely related to anti-neuroinflammation, anti-oxidative stress, inhibition of neuronal apoptosis, and protection of the cholinergic system (Dan et al., 2023). While AD pathophysiology differs from MS, this robust preclinical evidence provides mechanistic support for BBR's neuroprotective potential and justifies further investigation in human trials.

In acute ischemic stroke, a randomized controlled trial by Li et al. (2016) involving 120 patients demonstrated that BBR (300 mg three times daily for 14 days) as an adjunct to standard therapy significantly reduced serum inflammatory markers (IL−6, MIF) and improved neurological outcomes (NIHSS scores) compared to control (Li et al., 2016). These findings are supported by a recent meta-analysis by Luo et al. (2023) that pooled data from 17 clinical trials (1670 patients) and confirmed that adjuvant BBR therapy significantly reduces inflammatory cytokines (IL−6, hs-CRP, MIF) and improves functional outcomes in acute ischemic stroke patients [citation: based on previous search]. This body of evidence suggests that BBR's anti-inflammatory effects observed in preclinical models may translate to humans with acute CNS injury. In schizophrenia, a randomized controlled trial by Li et al. (2022) demonstrated that adjunctive BBR treatment (400 mg/day for 8 weeks) significantly improved negative symptoms and reduced serum pro-inflammatory cytokines, with good tolerability. This study confirms BBR's immunomodulatory effects in humans and supports its safety in combination with other CNS-active medications. In metabolic disorders, extensive clinical research has established BBR's efficacy in improving glycemic control, lipid profiles, and insulin sensitivity (Choi et al., 2006, Zhang et al., 2020). These findings are relevant to MS because cardiovascular risk factors may influence disease progression and neuroinflammation.

In summary, while BBR's preclinical profile in MS models is compelling, its clinical potential remains unrealized due to significant pharmacokinetic barriers and the complete absence of human studies. The promising results with BBR-IONP in the CPZ model (Ibrahim Fouad et al., 2024) provide a foundation for future nanoformulation research, but systematic translational studies are urgently needed to determine whether BBR can fulfill its therapeutic promise in MS patients. Future research should prioritize: (1) optimization of CNS-targeted nanoformulations; (2) comprehensive toxicology and biodistribution studies; (3) phase I clinical trials to establish safety and pharmacokinetics; and (4) proof-of-concept trials in MS patients to evaluate preliminary efficacy on clinical and radiographic outcomes.

### 4.8 Additional pathways with potential relevance to MS: insights from non-MS models

Beyond the mechanisms directly validated in EAE and CPZ models, several other signaling pathways—well-characterized in general BBR biology and non-MS disease models—may contribute to BBR's therapeutic potential in MS. While these pathways await direct confirmation in MS-specific models, their established roles in neuroinflammation, oxidative stress, and immune modulation warrant discussion.

#### 4.8.1 NF-κB pathway

The NF-κB pathway is a central mediator of inflammatory responses and has been extensively studied in the context of BBR's anti-inflammatory effects. In general, BBR has been shown to inhibit NF-κB activation by preventing the degradation of IκBα (inhibitor of κB), thereby reducing nuclear translocation of p65 and subsequent transcription of pro-inflammatory genes (Shou and Shaw, 2022, Kuo et al., 2012). This mechanism has been demonstrated in various non-MS models, including collagen-induced arthritis (Vita et al., 2021), experimental autoimmune uveitis (Du et al., 2020), and experimental autoimmune myocarditis (Liu et al., 2016). Given the pivotal role of NF-κB in MS-associated neuroinflammation—particularly in microglial activation and cytokine production—this pathway represents a plausible target for BBR in MS, though direct evidence from EAE or CPZ models remains limited.

#### 4.8.2 Nrf2/HO−1 pathway

The Nrf2/HO−1 pathway is a primary cellular defense mechanism against oxidative stress. BBR has been demonstrated to activate this pathway in multiple non-MS contexts, including models of neurodegenerative diseases (Cheng et al., 2022, Sunhe et al., 2024). Activation of Nrf2 leads to upregulated expression of antioxidant enzymes such as HO−1, NAD(P)H: quinone oxidoreductase 1 (NQO1), and glutathione S-transferase (GST), thereby reducing reactive oxygen species (ROS) and protecting against oxidative damage (Imenshahidi and Hosseinzadeh, 2020, García-Muñoz et al., 2024). In the context of MS, oxidative stress contributes significantly to oligodendrocyte injury and axonal damage. While direct evidence for Nrf2/HO−1 activation by BBR in MS models is currently lacking, the demonstrated neuroprotective effects of BBR in the CPZ model—including reduced oxidative stress markers and increased glutathione (GSH) levels (Ibrahim Fouad et al., 2024)—are consistent with Nrf2 pathway activation and warrant further investigation.

#### 4.8.3 AMPK signaling

AMP-activated protein kinase (AMPK) is a central regulator of cellular energy homeostasis and has emerged as a key target in both metabolic and inflammatory disorders. BBR is a well-established AMPK activator, and this mechanism underlies many of its metabolic benefits in diabetes and metabolic syndrome (Lee et al., 2006, Brusq et al., 2006, Mbara et al., 2025). AMPK activation by BBR has been shown to suppress mTOR signaling, enhance autophagy, and reduce inflammatory responses in various cell types (Och et al., 2020). In the context of MS, AMPK activation may contribute to neuroprotection by promoting mitochondrial function, reducing ER stress, and modulating microglial polarization toward an anti-inflammatory (M2) phenotype. However, direct evidence linking BBR-induced AMPK activation to therapeutic effects in EAE or CPZ models has not yet been reported.

#### 4.8.4 PI3K/Akt pathway

The phosphatidylinositol 3-kinase (PI3K)/Akt pathway is a critical mediator of cell survival, proliferation, and differentiation. In non-MS models, BBR has been shown to modulate this pathway in a context-dependent manner—activating it to promote neuronal survival in models of neurodegenerative diseases (Chen et al., 2012, Akbar et al., 2021), while inhibiting it to suppress proliferation in cancer models (Kuo et al., 2012, Goel, 2023). This bidirectional regulation highlights the complexity of BBR's effects. In MS, PI3K/Akt signaling may influence oligodendrocyte survival, axonal integrity, and remyelination. While direct evidence from MS models is lacking, the pro-remyelinating effects observed with BBR-IONP in the CPZ model (Ibrahim Fouad et al., 2024) are consistent with potential involvement of survival pathways and merit further mechanistic exploration.

#### 4.8.5 Summary of pathway evidence in MS context

In summary, several pathways have been directly validated in MS-relevant models. Qin et al (Qin et al., 2010). established the role of JAK/STAT signaling in BBR's immunomodulatory effects, demonstrating suppression of Th1 and Th17 differentiation in EAE. Luo et al. (2017) confirmed that BBR targets the SPHK1/S1P pathway, reducing demyelination and astrocyte activation in both CNS and peripheral compartments. Jiang et al. (2013) provided evidence for MMP−9 inhibition and preservation of blood-brain barrier integrity. Beyond these, additional pathways with strong theoretical relevance to MS—including NF-κB, Nrf2/HO−1, AMPK, and PI3K/Akt—have been extensively characterized in non-MS models.

Beyond these, additional pathways with strong theoretical relevance to MS—including NF-κB (Shou and Shaw, 2022), Nrf2/HO−1 (Cheng et al., 2022, Akbar et al., 2021), and PI3K/Akt (Cheng et al., 2022; Akbar et al., 2021)—have been extensively characterized in non-MS models but await direct confirmation in EAE or CPZ systems. Future mechanistic studies should prioritize direct investigation of these pathways in MS-specific models to determine whether they contribute to BBR's therapeutic effects and to identify optimal combinations for multi-targeted intervention.

### 4.9 Critical appraisal of safety in the MS context

While BBR demonstrates promising therapeutic effects in preclinical MS models, a balanced evaluation of its safety profile is essential, particularly for the MS population who may receive concurrent immunomodulatory therapies.

BBR is generally regarded as safe for short-term use in healthy adults, with common adverse effects being mild gastrointestinal disturbances (nausea, diarrhea, constipation) that are dose-dependent and often resolve with continued use (Qin et al., 2024, Kumar et al., 2015). However, comprehensive long-term safety data remain lacking, and no studies have systematically evaluated BBR's safety specifically in MS patients. A critical consideration for MS applications is emerging evidence of BBR's biphasic effects on neuronal viability. Studies have demonstrated that low micromolar concentrations (>1 μM) can cause mitochondrial toxicity, ATP depletion, and NMDA receptor-dependent cell death in primary neurons. Importantly, subtoxic nanomolar concentrations—comparable to achievable plasma levels—may sensitize neurons to glutamate excitotoxicity and oxidative injury (Kysenius et al., 2014).

BBR is contraindicated with cyclosporine, as studies have shown that BBR can increase cyclosporine blood concentrations (Colombo et al., 2014). Additionally, BBR inhibits CYP2D6 and CYP3A4 (Bathaei et al., 2025), which may affect the metabolism of concomitant medications. BBR is also a substrate of P-glycoprotein, potentially altering the disposition of co-administered P-gp substrates. Its immunomodulatory effects could theoretically augment or interfere with disease-modifying therapies (DMTs) (fingolimod, natalizumab, ocrelizumab), though specific interaction data are lacking and require investigation. In addition, BBR should be avoided during pregnancy and lactation due to several well-documented risks. BBR can cross the placental barrier and has been shown to displace bilirubin from albumin with approximately tenfold greater potency than phenylbutazone, a known potent displacer. This displacement can lead to elevated free bilirubin levels in neonates, increasing the risk of kernicterus (bilirubin-induced brain damage) in jaundiced infants (Chan, 1993). While nanoformulations enhance BBR bioavailability, they introduce additional safety considerations. Recent studies show that nanostructured lipid carriers (NLCs) themselves can increase microsomal malondialdehyde (MDA) and comet formation, indicating potential oxidative stress and genotoxicity (Stefanova et al., 2025).

A critical safety consideration for MS patients is the potential for additive immunosuppression when BBR is combined with DMTs. BBR inhibits CYP3A4 and CYP2D6, enzymes crucial for metabolizing many immunosuppressants (Bathaei et al., 2025). Specifically, BBR is contraindicated with cyclosporine due to documented interactions affecting drug levels (Khoshandam et al., 2022). While BBR's immunomodulatory effects—suppression of Th1/Th17 and JAK/STAT inhibition (Liu et al., 2016). Additionally, BBR's effects on vaccine response and immune reconstitution after DMT cessation remain unknown. Until dedicated drug interaction studies are available, caution and careful monitoring (lymphocyte counts, infection surveillance) are warranted when considering BBR as an adjunct to DMTs (Kumar et al., 2015).

## 5 Conclusion

In conclusion, BBR represents a promising therapeutic candidate for MS based on robust preclinical evidence demonstrating its ability to target key pathological processes, including immune dysregulation, BBB disruption, demyelination, and neurodegeneration. However, it is essential to emphasize that no clinical trials have yet evaluated BBR in MS patients. The translational gap between preclinical promise and clinical application remains substantial, primarily due to pharmacokinetic barriers and the absence of human data. Future research must prioritize the development of optimized nanoformulations to enhance bioavailability and CNS delivery, followed by rigorous clinical trials to establish safety and efficacy in the MS population. Only through such systematic translational research can the therapeutic potential of BBR be realized for individuals living with multiple sclerosis.

## CRediT authorship contribution statement

**Razie Omrani:** Writing – original draft. **Hediyeh Dasoomi:** Writing – original draft. **Mohammadhadi Nikjoo:** Writing – original draft. **Reza Arefnezhad:** Writing – review & editing, Writing – original draft. **Amir Modarresi Chahardehi:** Writing – original draft, Data curation. **Zahra Jafari-Ardakan:** Writing – review & editing. **Narges Moazeni Limoudehi:** Writing – original draft. **Fatemeh Rezaei-Tazangi:** Supervision, Project administration. **Negar Karimi Khordeh:** Writing – original draft. **Reza Nasiri:** Writing – original draft. **Elham Mortazavi Mamaghani:** Writing – original draft. **Fatemeh Shoja:** Writing – original draft. **Mohammad Saeed Soleimani Meigoli:** Writing – original draft.

## Informed consent

Not applicable.

## Ethics approval

Ethical issues (including plagiarism, data fabrication, double publication) have been completely observed by the authors.

## Funding

This work was not financially supported.

## Declaration of Competing Interest

The authors declare no competing interests.

## Data Availability

The datasets used and/or analyzed during the current study are available from the corresponding author upon reasonable request.
